# Fulvestrant in the treatment of hormone receptor‐positive/human epidermal growth factor receptor 2‐negative advanced breast cancer: A review

**DOI:** 10.1002/cam4.2095

**Published:** 2019-04-19

**Authors:** Junjie Li, Zhonghua Wang, Zhimin Shao

**Affiliations:** ^1^ Department of Surgery in Breast Cancer Fudan University Shanghai Cancer Center Shanghai China

**Keywords:** advanced breast cancer, fulvestrant, HER2−, HR+, targeted therapies

## Abstract

Nearly 75% of breast cancers are hormone receptor‐positive (HR+) and human epidermal growth factor receptor type 2‐negative (HER2−), making endocrine therapy the mainstay of treatment for HR+ and HER2− combination. Although endocrine therapy, such as therapy with fulvestrant, is widely used in the clinic, endocrine resistance (primary or secondary) is inevitable and poses a serious clinical concern. However, the therapeutic landscape of HR+/HER2− breast cancer is rapidly changing and evolving. In recent years, molecular insights into the genome of HR+/HER2− breast cancer have helped to identify promising targets, such as alterations in signaling pathways [phosphatidylinositide 3‐kinase (PI3K/AKT/mammalian target of rapamycin (mTOR)], dysregulation of the cell cycle (CDK4/6), and identification of new *ESR1* mutations. These insights have led to the development of newer targeted therapies, which aims at significantly improving survival in these patients. This review summarizes the role and rationale of fulvestrant when used as a monotherapy or in combination with targeted therapies in patients with HR+/HER2− advanced breast cancer. We also discuss other novel agents and potential future combination treatment options.

## 1 INTRODUCTION

Breast cancer (BC) is a heterogeneous disease, comprising multiple subgroups of varying molecular signatures, prognoses, and responses to therapies.^1^ From the clinical perspective, BC can be subdivided into three major subtypes: tumors expressing estrogen receptor (ER) and/or progesterone receptor (PgR; commonly referred to as hormone receptor‐positive [HR+]), ErbB2‐amplified (also known as human epidermal receptor 2‐amplified [HER2+]), and triple‐negative BC (TNBC) due to the absence of ER/PgR and normal or negative HER2 expression.^2^

Postmenopausal women are highly predisposed to BC, and about 67% to 70% of all reported metastatic BCs are HR+, which are potentially sensitive to endocrine therapy.^3^ The treatment for HR+/HER2− locally advanced or metastatic BC is largely palliative, mostly aiming at prolonging survival and/or to improve or at least maintain quality of life and delay the initiation of chemotherapy.^4^ Selection of treatment is mainly based on four factors: the extent of disease, prior response to adjuvant endocrine therapy, the patient's clinical status, and patient preference.^4^ As per major international guidelines, endocrine therapy is regarded as the cornerstone treatment for HR+/HER2− advanced BC and should be considered for the majority of patients with locally advanced or metastatic tumors, with exceptions for those with life‐threatening disease, those experiencing visceral crisis, or those with prior endocrine resistance.^5^, ^6^, ^7^

Endocrine therapies for the treatment of HR+/HER2− advanced BC include tamoxifen, the selective estrogen receptor modulator; nonsteroidal and steroidal aromatase inhibitors (AIs), which inhibit the peripheral synthesis of estrogen, thereby reducing estrogen levels (eg, anastrozole, letrozole, and exemestane); fulvestrant, the selective estrogen receptor downregulator (SERD).^5^, ^8^

The therapeutic field of cancer therapy has been constantly expanding in recent years, offering newer and potentially more effective agents. Insights into molecular and biological pathways that may contribute to endocrine resistance has led to the approval of several targeted agents, viz., mammalian target of rapamycin (mTOR) and cyclin‐dependent kinase 4, 6 (CDK4/6) inhibitors. For example, the use of mTOR inhibitor, everolimus, or CDK4/6 inhibitor, palbociclib, in combination with endocrine therapy has proven to be among the most crucial advances in the management of HR+/HER2− advanced BC over the last 5 years.^9^ Since the development of these agents, further combinations of targeted drugs and endocrine therapies have been clinically approved.^10^, ^11^ However, the optimal choice and sequence of endocrine therapies is not clearly defined.

Given the evolving role of fulvestrant in the management of BC, this manuscript aims to review its clinical efficacy data and current role in the systemic therapy of advanced or metastatic HR+/HER2− BC as monotherapy or in combination with other therapeutic modalities.

## 2 FULVESTRANT: MECHANISM OF ACTION

**Figure 1:**
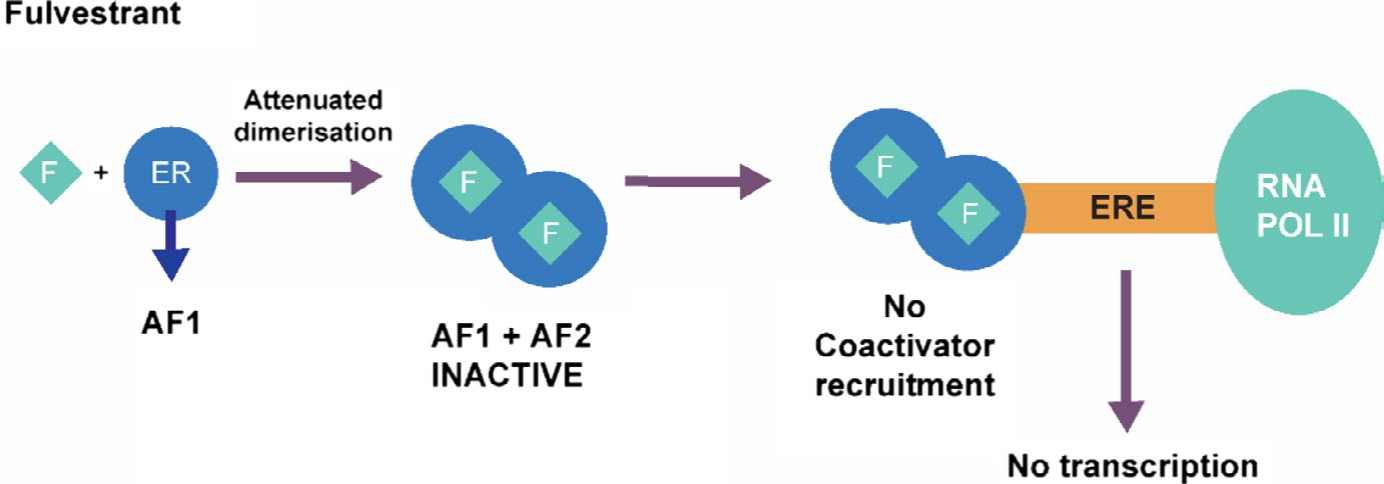
A schematic representation of the action of fulvestrant. AF1, activation function 1; AF2, activation function 2; ER, estrogen receptor; ERE, estrogen receptor response element; F, fulvestrant; RNA POL II, ribonucleic acid polymerase II. Adapted from Boer, Ther. Adv. Med. Oncol. 2017;9(7)465‐479^4^

Fulvestrant exerts selective ER downregulation via binding competitively to ERBinding of fulvestrant to ER inhibits ER receptor dimerization, blocking nuclear localization of the receptor.^12^, ^13^ Fulvestrant is shown to have a binding affinity, which is 100 times greater than the affinity of other endocrine drug class, tamoxifen.^14^, ^15^ Binding of fulvestrant to ER also leads to a rapid degradation of the fulvestrant‐ER complex, making the receptor unavailable to estrogen and attenuating the ability of ER to promote gene transcription.^16^ Another characteristic of fulvestrant, which distinguishes its mode of action from that of tamoxifen, is that it consistently reduces estrogen and PgR levels in tumor cells, without having agonist effects.^17^, ^18^ Figure 1 shows a schematic representation of the mode of action of fulvestrant.

## 3 FULVESTRANT AS MONOTHERAPY

Several trials have assessed the efficacy of fulvestrant as a single agent in HR+/HER2− advanced BC. Initial studies have demonstrated fulvestrant's non‐inferiority compared with tamoxifen.^19^, ^20^ In a multicenter, double‐blinded, randomized trial, patients with metastatic/locally advanced BC previously untreated for advanced disease were randomly assigned to either the fulvestrant (250 mg, via intramuscular injection, once monthly; n = 313) group or tamoxifen (20 mg, orally, once daily; n = 274) group. There was no significant difference between these two endocrine therapies in terms of time to progression (TTP).^19^ Likewise, another double‐blind randomized trial compared the efficacy and tolerability of fulvestrant (n = 206) vs anastrozole (n = 194) in postmenopausal women with advanced BC who were progressing even after prior endocrine therapy. It was found that fulvestrant was as effective as anastrozole in terms of efficacy endpoints.^21^

Although fulvestrant 250 mg is adequate to competitively inhibit binding of estradiol to ER, inhibition of ER transcription may occur without a complete degradation of receptor.^12^ In continuation, supporting evidence showing the effect of fulvestrant dose on efficacy in patients with HR+/HER2− advanced BC began emerging. Results from FINDER 1 (NCT00305448) and 2 (NCT00313170) have consistently shown that in Western and Japanese postmenopausal ER+ locally advanced/metastatic BC patients, a high dose (500 mg) of fulvestrant had similar or improved efficacy and tolerability compared with the approved dose of 250 mg.^22^, ^23^ Fulvestrant 500 mg/month and fulvestrant 250 mg/month was compared in ER+ advanced BC women in Comparison of Faslodex in Recurrent or Metastatic Breast Cancer (CONFIRM; NCT00099437), a randomized, double‐blind, phase III trial.^24^ It was observed that progression‐free survival (PFS) was significantly longer with 500 mg (n = 362) compared with 250 mg (n = 374; hazard ratio, 0.80; 95% class interval [CI], 0.68‐0.94; *P* = 0.006). The initial analysis showed a 20% reduction in the risk of progression with fulvestrant 500 mg and a nonsignificant difference of 2.3 months in median overall survival (OS) compared with fulvestrant 250 mg (25.1 months vs 22.8 months; hazard ratio, 0.84; 95% CI, 0.69‐1.03; *P* = 0.09).^24^ A final analysis of OS for this trial showed that fulvestrant 500 mg was associated with 19% reduction in risk of death and a 4.1 months difference in median OS compared with fulvestrant 250 mg (26.4 vs 22.3 months; hazard ratio, 0.81; 95% CI, 0.69‐0.96; *P* = 0.020).^25^ A post hoc analysis of the CONFIRM trial also showed that first‐line fulvestrant (500 mg) significantly prolonged PFS compared with fulvestrant 250 mg (median PFS 5.6 vs 4.2 months; hazard ratio, 0.80; 95% CI, 0.64‐1.00; *P* = 0.047). As second‐line therapy, PFS with fulvestrant 500 mg was numerically greater than fulvestrant 250 mg (7.9 vs 6.3 months; hazard ratio, 0.80; 95% CI, 0.64‐1.02; *P* = 0.068). Median OS with first‐line fulvestrant 500 mg was 23.2 vs 22.1 months with fulvestrant 250 mg (hazard ratio, 0.87; 95% CI, 0.70‐1.10; *P* = 0.251) and that with second‐line it was 29.2 vs 22.8 months (hazard ratio, 0.75; 95% CI, 0.58‐0.96; *P* = 0.020).This suggested superiority of higher doses of fulvestrant in both first‐ and second‐line settings.^26^

Yet another phase II, randomized, open‐label study FIRST (NCT01602380) was designed to evaluate fulvestrant 500 mg (n = 102) in comparison with anastrozole 1 mg (n = 103) as first‐line endocrine therapy for postmenopausal women with HR+/HER2− advanced BC.^27^ The primary outcome of interest for this non‐inferiority trial was clinical benefit rate (CBR), which was similar for fulvestrant and anastrozole (72.5% vs 67.0%; odds ratio, 1.30; 95% CI, 0.72‐2.38; *P* = 0.385). TTP was significantly longer for fulvestrant compared with anastrozole (median TTP not reached for fulvestrant vs 12.5 months for anastrozole; hazard ratio, 0.63; 95% CI, 0.39‐1.00; *P* = 0.049).^27^ Results of a follow‐up analysis of this trial showed a median TTP of 23.4 months for the fulvestrant group vs 13.1 months for the anastrozole group (hazard ratio, 0.66; 95% CI, 0.47‐0.92; *P* = 0.010) corresponding to a 34% reduction in risk of progression.^28^ Furthermore, in this trial, OS analysis was planned when approximately 65% of patients had died and it was observed that OS was extended with fulvestrant 500 mg compared with anastrozole (54.1 vs 48.4 months; hazard ratio, 0.70; 95% CI, 0.50‐0.98; *P* = 0.040).^29^

Based on these findings, the potential benefits of fulvestrant 500 mg was investigated in fulvestrant and Anastrozole compared in hormonal therapy‐naïve advanced breast cancer (FALCON; NCT01602380), which was a randomized, double‐blind, multicenter phase III trial.^30^ Fulvestrant 500 mg (n = 230) showed a PFS of 16.6 months vs 13.8 months with anastrozole 1 mg/day (n = 232) (hazard ratio, 0.797; 95% CI, 0.637‐0.999; *P* = 0.048). A 21% reduction in risk of disease progression or death in women with locally advanced or metastatic HR+/HER2− BC who had been treated with fulvestrant 500 mg compared with those who received anastrozole 1 mg/day was observed.^31^

To summarize, fulvestrant 500 mg offers greater antitumor activity than the 250‐mg regimen,^32^, ^33^ without showing significant differences in toxicity profile. As per the US Food and Drug Administration (FDA) and European Medicine Agency (EMA) product information, fulvestrant has been approved and indicated in treating postmenopausal women with ER+, advanced or metastatic BC, either for disease relapse, on or after adjuvant antiestrogen therapy or for disease progression following endocrine therapy.^34^, ^35^ In China, fulvestrant has been launched as first‐line therapy for late‐stage BC, metastatic disease.^36^

## 4 PROGNOSTIC BIOMARKERS OF FULVESTRANT THERAPY

Although several mechanisms have been elucidated to understand endocrine resistance, no particular biomarker or gene signature has been attributed; particularly for clinical use. In these lines, numerous multi‐gene expression based assays have been developed to assess response to fulvestrant treatment and chemotherapy in early stage ER + BC.

It is found that mRNA levels of TFAP2C, a transcription factor expressed in BC, correlated with the protein expression levels and high transcript and protein levels correlated with decreased response to fulvestrant treatment.^37^ Interestingly, primary tumors from a subset of patients enrolled in the CONFIRM trial were evaluated in the transCONFIRM study in order to recognize a gene signature of response to fulvestrant in advanced BC. It was reported that increased epidermal growth factor (EGF) pathway and Forkhead box protein A1 (FOXA1) transcriptional signaling were associated with a decreased response to fulvestrant. Furthermore, the reduced response to fulvestrant was attributed to a set of 37 genes with an expression pattern independently associated with PFS that demonstrated high expression of the *TFAP2C* gene (a well‐known regulator of ER activity). The negative predictive value of TFAP2C expression, therefore, suggests further validation of fulvestrant treatment as a predictive biomarker in metastatic BC.^37^

Additionally, analysis of mutations in ER gene (ESR1), mostly found in patients progressing after prior AIs are also gathering attention. A prospective‐retrospective analysis of SoFEA trial demonstrated that patients with ESR1 mutations predicted relative sensitivity to fulvestrant but resistance to exemestane.^38^ On the contrary, a recent meta‐analysis showed no association between ESR1 mutation status and fulvestrant efficacy.^39^ In this context, further comprehensive studies reporting a possible gene signature to efficiently predict response to fulvestrant therapy and aid clinical decisions are needed.

## 5 FULVESTRANT IN COMBINATION WITH OTHER ENDOCRINE THERAPIES

Fulvestrant and Anastrozole Combination Therapy (FACT; NCT00256698) was an open‐label, randomized, phase III trial which reported no clinical benefit by combining fulvestrant 250 mg plus anastrozole vs anastrozole monotherapy.^40^ On the contrary, in the Southwest Oncology Group (SWOG; NCT00075764) open‐label, randomized, phase III trial, the results favored this combination approach over anastrozole alone.^41^ In this study, median PFS among women who had not received prior tamoxifen therapy was 12.6 months with anastrozole monotherapy compared with 17.0 months as seen in the combination arm (hazard ratio, 0.74; 95% CI, 0.59‐0.92; *P* = 0.006).^41^ It is noteworthy to add that while the addition of fulvestrant to anastrozole improved OS in postmenopausal patients with HR + metastatic BC in the SWOG trial, a pharmacokinetic interaction has been suggested to occur wherein fulvestrant decreases anastrozole concentrations and persists throughout treatment.^42^ Although the clinical relevance of this interaction is unclear, addition of fulvestrant to anastrozole may compromise the efficacy of anastrozole. A summary of studies investigating fulvestrant as a single agent or in combination with other endocrine and/or targeted therapies for the treatment of HR + advanced BC is given in Table 1.

**Table 1:** List of phase II/III trials using fulvestrant

Study/Trial name (n)	Treatment regimen	Line of treatment	CBR/ORR (%)	Survival	
				OS, months	PFS, months
Fulvestrant as monotherapy					
FALCON (n = 462)^31^	F 500 mg, anastrozole 1 mg	1st	CBR: 78 vs 74	NA	16.6 vs 13.8*
FIRST (n = 205)^27^, ^28^, ^29^	F 500 mg, anastrozole 1 mg	1st	CBR: 72.5 vs 67.0	54.1 vs 48.4*	23.4 vs 13.1*
CONFIRM (n = 736)^24^, ^25^	F 500 mg, F 250 mg	2nd	CBR: 45.6 vs 39.6	26.4 vs 22.3*	6.5 vs 5.5**
Fulvestrant + other endocrine therapy					
FACT (n = 514)^40^	F 250 mg + anastrozole, anastrozole	1st	NA	38.2 vs 37.8	10.8 vs 10.2
SWOG (n = 694)^41^	F 250 mg + anastrozole, anastrozole, fulvestrant	1st	CBR: 73.0 vs 70.0	47.7 vs 41.3*	15 vs 13.5**
SoFEA (n = 723) 43	F 500/250 mg + anastrozole, F 500 mg + placebo, exemestane	2nd	NA	20.2 vs 19.4	4.4 vs 4.8 vs 3.4
Fulvestrant + CDK inhibitor					
PALOMA 3 (n = 521)^44^, ^45^	F 500 mg + palbociclib, F 500 mg + placebo	2nd	CBR: 24.6 vs 10.9*	NA	9.5 vs 4.6**
MONARCH 2 (n = 669)^46^	F 500 mg + abemaciclib vs F 500 mg + placebo	2nd	ORR: 48.1 vs 21.3	NA	16.4 vs 9.3*
MONALEESA 3 (n = 726)^47^	F 500 mg + ribociclib. F 500 mg + placebo	2nd	ORR: 32.4 vs 21.5**	NA	20.5 vs 12.8**
Fulvestrant + mTOR inhibitor					
PrECOG (n = 131)^48^	F 500 mg + everolimus, F 500 mg + placebo	2nd	NA	NA	10.4 vs 5.1*
Fulvestrant + PI3K inhibitor					
SANDPIPER (n = 516)^49^	F 500 mg + taselisib, F 500 mg + placebo	2nd	ORR: 28.0 vs 11.9** CBR: 51.5 vs 37.3	NA	7.4 vs 5.4*
BELLE‐2 (n = 1147)^50^	F 500 mg + buparlisib, F 500 mg + placebo	2nd	ORR: 11.8 vs 7.7	NA	6.9 vs 5.0**
FERGI (n = 168)^51^	F 500 mg + pictilisib, F 500 mg + placebo	2nd	ORR: 7.9 vs 6.3	NA	6.6 vs 5.1
BELLE‐3 (n = 432)^52^	F 500 mg + buparlisib, F 500 mg, F 500 mg + placebo	2nd	ORR: 7.6 vs 2.1	7.6 vs 2.1	3.9 vs 1.8**
LEA (n = 380)^53^	F 250 mg or letrozole + bevacizumab, F 250 mg or letrozole + placebo	1st	CBR: 76.8 vs 67.4	52.1 vs 51.8	19.3 vs 14.4
Fulvestrant + EGFR, HER2 inhibitor					
CALGB (n = 291)^54^	F 500 mg + lapatinib, F 500 mg + placebo	1st	NA	30 vs 26.4	4.7 vs 3.8
Robertson et.al (n = 156)^55^	F 250 mg or exemestane + ganitumab, F 250 mg or exemestane + placebo	2nd	NA	22.2 vs NA	5.7 vs 3.9

CDK, cyclin‐dependent kinase; CBR, clinical benefit rate; ORR, overall response rate; EGFR, epidermal growth factor receptor; F, fulvestrant; HER2, human epidermal growth factor receptor type 2; IGFR, insulin‐like growth factor receptor; mTOR, mammalian target of rapamycin; N, number of patients; OS, overall survival; PFS, progression‐free survival; PI3K, phosphoinositide 3‐kinase. Modified from Boer, Ther. Adv. Med. Oncol. 2017;9(7):465‐479.^4^

* *P* < 0.05.

** *P* < 0.001.

## 6 FULVESTRANT IN COMBINATION WITH TARGETED THERAPIES

**Figure 2:**
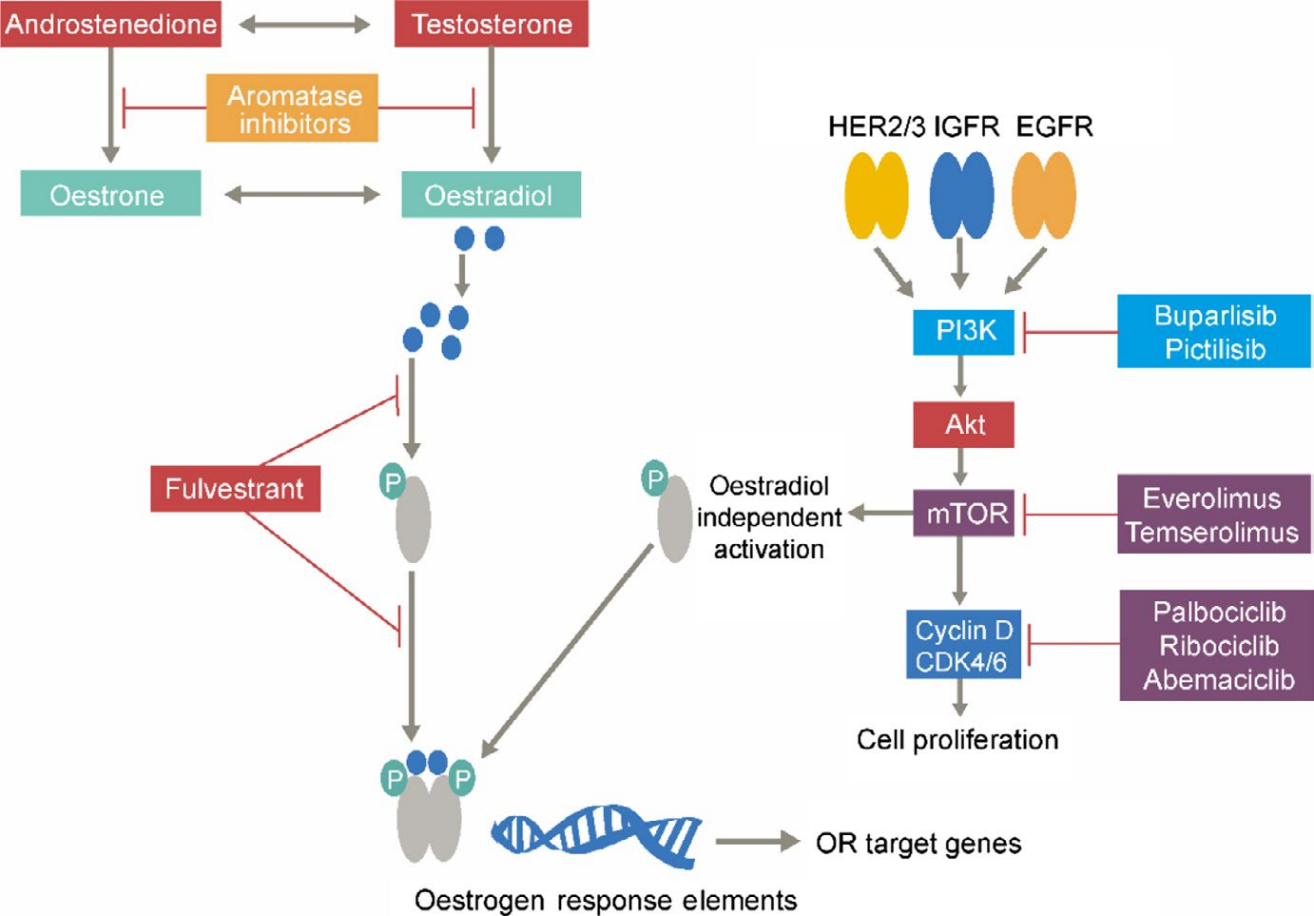
Mode of action of fulvestrant and other targeted therapies in cancer cells. AKT, protein kinase B; CDK4/6, cyclin‐dependent kinases 4/6; EGFR, epidermal growth factor receptor; HER2/3, human epidermal growth factor receptor 2/3; IGFR, insulin‐like growth factor receptor; mTOR, mammalian target of rapamycin; PI3K, phosphatidylinositol‐3‐kinase; OR, estrogen receptor. Adapted from Peter, Eur. Oncol. Haematol., 2017;13(2):127‐133^58^

Although endocrine monotherapy is successful in treating majority of patients with HR+/HER2− BC, a significant number of cases do report a relapse and become refractory to such approaches.^25^ Reasons for such resistance can be attributed to several factors, including: activating mutations in the *ESR1* gene that encodes ER, increased activity of CDK4/6, upregulation of signaling pathways such as phosphoinositide‐3‐kinase (PI3K)/AKT/mTOR and HER2/mitogen‐activated protein kinase (MAPK).^5^, ^56^, ^57^ Tapping these potential molecular and genomic alterations leading to endocrine resistance has resulted in development of targeted therapies, changing the landscape of HR+/ HER2− advanced BC treatment (Figure 2).

The combination of fulvestrant with targeted agents is evolving, with clinical trials targeting the aforementioned signaling mechanisms, thereby increasing or restoring endocrine sensitivity (Figure 2). Results from phase III trials have favored the combination of AIs with mTOR inhibitor, everolimus, and CDK 4/6 inhibitors (abemaciclib, palbociclib, and ribociclib), by providing superior efficacy in patients who have previously received an AI^38^, ^59^ and thus have been approved for metastatic HR + BC.^47^, ^60^, ^61^, ^62^

### 6.1 Fulvestrant in combination with CDK 4/6 inhibitor

Inhibiting increased activity of CDK4/6 in HR + BC establishes a new therapeutic strategy to enhance the efficacy of fulvestrant therapy and also potentially reverse fulvestrant resistance.^44^. Among three specific CDK4/6 inhibitors, palbociclib, abemaciclib, and ribociclib, palbociclib was the first drug in its class that was introduced into clinical practice.^45^, ^60^

Preclinical investigation of palbociclib, a selective CDK4/6 inhibitor (Figure 2), present a strong rationale for clinical studies to test the combination of palbociclib with fulvestrant in HR+/HER2− BC patients.

A multicenter, double‐blind, randomized phase III PALOMA 3 study (n = 521; NCT01942135) investigated the combination of fulvestrant and palbociclib as second‐line treatment for patients with HR+/HER2− advanced BC. Eligible women with any menopausal status (pre‐ or perimenopausal who were administered with any luteinizing hormone‐releasing hormone at least 4 weeks before study therapy initiation) who relapsed or progressed during or <12 months of endocrine therapy or while on or <1 month from prior endocrine therapy for the condition were included.^63^, ^64^ It was seen that combination therapy of fulvestrant with palbociclib showed a significant and consistent improvement in PFS compared with fulvestrant plus placebo (median 9.2 vs 3.8 months, respectively; hazard ratio for disease progression or death, 0.42; 95% CI, 0.32‐0.56; *P* < 0.001), irrespective of the degree of endocrine resistance, HR expression level, and PIK3CA mutational status.^45^, ^63^ The median OS was 34.9 months in palbociclib‐fulvestrant group and 28.0 months in the placebo‐fulvestrant group (hazard ratio for death, 0.81; 95% CI, 0.64‐1.03; *P* = 0.09; absolute difference, 6.9 months). On the other hand, the median OS in patients with sensitivity to previous endocrine therapy (n = 410) was 39.7 months in the palbociclib‐fulvestrant group and 29.7 months in the placebo‐fulvestrant group (hazard ratio, 0.72; 95% CI, 0.55‐0.94; absolute difference, 10.0 months).^65^ Treatment with this combination was generally safe and well tolerated, with neutropenia representing the most common adverse event. Unlike results seen with chemotherapy, despite the high rate of grade 3 to 4 neutropenia (62%), the rate of febrile neutropenia was very low (0.6%) in the palbociclib arm. Overall, these data show promising efficacy with fulvestrant plus palbociclib, with manageable adverse events.^44^ Based on these data, the FDA and European Union have approved the use of fulvestrant 500 mg for treating HR+/HER2− advanced or metastatic BC in combination with palbociclib in women with disease progression following endocrine therapy.^66^, ^67^

Abemaciclib, another inhibitor of Rb phosphorylation, has been found to inhibit tumor growth in mouse models.^68^ This has been investigated in combination with fulvestrant in the MONARCH 2 (NCT02107703) study. This global, double‐blind, phase III study compared PFS among patients receiving abemaciclib plus fulvestrant (n = 446) vs fulvestrant alone (n = 223) in HR+/HER2− advanced BC patients who were aged ≥18 years, whose disease progressed while receiving prior endocrine therapy. Eligible women with any menopausal status (pre‐ or perimenopausal women who received a gonadotropin‐releasing hormone agonist) and had an Eastern Cooperative Oncology Group performance status of 0 or 1 were enrolled. Additionally, patients were required to have disease progression while on neoadjuvant or adjuvant endocrine therapy for ≤12 months after adjuvant or while receiving endocrine therapy for advanced BC. The combination of abemaciclib plus fulvestrant significantly increased PFS compared with fulvestrant monotherapy (median 16.4 vs 9.3 months; hazard ratio, 0.553; 95% CI, 0.449‐0.681; *P* < 0.001).^46^ Unlike palbociclib, the most common adverse event in the abemaciclib arm was diarrhea (86.4%), followed by neutropenia (46.0%), nausea (45.1%), and fatigue (39.9%).

Results from the latest MONALEESA 3 trial (NCT02422615), a phase III, double‐blind, placebo‐controlled international study, also corroborates the above findings with respect to ribociclib. Postmenopausal women and men with histologically and/or cytologically confirmed HR+/HER2− advanced BC were included in the study. Patients were required to have advanced BC that was newly diagnosed, thus receiving first‐line therapy or those who relapsed >12 months or ≤12 months from completion of (neo) adjuvant endocrine therapy. Additionally, patients who relapsed after >12 months from completion of (neo) adjuvant therapy with subsequent progression after one line of endocrine therapy for advanced or metastatic disease were also included. The study demonstrated favorable PFS with ribociclib plus fulvestrant combination (n = 210) compared with placebo (n = 151) (median 20.5 vs 12.8 months; hazard ratio, 0.59; 95% CI, 0.48‐0.73; *P* < 0.001) in HR+/HER2− advanced BC patients.^47^ Neutropenia (46.6%) was the common adverse event in the ribociclib arm, followed by leukopenia (13.5%), anemia (3.1%), fatigue (1.7%), and nausea (1.4%). Neutropenia was the only grade 4 adverse event reported in ≥5% of patients.^47^ Based on the results from this study, FDA recently approved the combination of ribociclib (KISQALI) with fulvestrant for treating postmenopausal women with HR+/HER2− advanced or metastatic BC, as initial endocrine‐based therapy or following disease progression on endocrine therapy.^69^ The list of ongoing phase II/III/IV trials combining CDK4/6 inhibitors with fulvestrant is summarized in Table 2.

**Table 2:** Key upcoming phase II/III/IV clinical trials combining CDK4/6 inhibitors with fulvestrant

Study name (ClinicalTrials.gov identifier)	Study arms	Study population	Outcomes measures
PADMA (NCT03355157)	Palbociclib + endocrine therapy vs chemotherapy with/without endocrine maintenance	Patients with metastatic HR+/HER2− BC in a real‐world setting	TTF
PEARL (NCT02028507)	Palbociclib + exemestane or fulvestrant vs capecitabine	Females with histologically confirmed metastatic BC whose disease is resistant to previous nonsteroidal AIs (letrozole or anastrozole) (on or within 12 months after end of adjuvant or within 1 month after end of endocrine treatment)	PFS and ORR
MAINTAIN (NCT02632045)	Ribociclib + fulvestrant vs fulvestrant + placebo	Patients with histologically or cytologically confirmed adenocarcinoma of the breast with unresectable or metastatic disease	PFS and ORR
PASIPHAE (NCT03322215)	Palbociclib + fulvestrant vs capecitabine	Patients with metastatic HR+/HER2− BC with progressive disease after endocrine treatment (on or within 12 months after end of adjuvant or within 1 month after end of endocrine treatment)	PFS, HRQOL, OS, and CBR
SONIA (NCT03425838)	AI + CDK4/6 (palbociclib/ribociclib) as first‐line therapy, followed by fulvestrant as second‐line therapy vs AI as first‐line therapy, followed by fulvestrant + CDK4/6 inhibitors in second‐line therapy	Women with HR+/HER2− advanced BC, who received prior treatment with an AI either as (neo)‐adjuvant or for advanced disease	PFS, OS, QOL, and ORR
PARSIFAL (NCT02491983)	Palbociclib + letrozole vs Palbociclib + fulvestrant	Aged ≥18 years or older, postmenopausal women with metastatic or locally advanced disease HR+/HER2− BC, not amenable to curative therapy. No prior chemotherapy line in the metastatic setting	PFS, TTP, OS,CBR, and ORR

AI, aromatase inhibitor; BC, breast cancer; CDK, cyclin dependent kinases; CBR, clinical benefit rate; HER2, human epidermal growth factor receptor 2; HR, hormone receptor; HRQOL, health related quality of life; OS, overall survival; ORR, overall response rate; PFS, progression‐free survival; QOL, quality of life; TTF, time to treatment failure; TTP, time to progression.

### 6.2 Fulvestrant in combination with pan‐PI3K inhibitors

Activation of the PI3K pathway is reported to be a hallmark of HR + BC cells that are resistant to endocrine therapy.^70^ PI3K pathway is frequently activated aberrantly in BC, with majority of mutations in the PI3K catalytic subunit (PIK3CA), encoding the catalytic p110α subunit.^71^ Approximately, 20%‐25% breast tumors exhibit these mutations depending on the BC subtype.^71^ Blocking ER and PIK3CA pathways therefore seems a promising strategy. *PI3KCA* mutations are frequently found in BC.^72^

However, clinical study results with pan PI3K inhibitors have been contradictory. The FERGI (NCT01437566) study, which was a randomized, double‐blind, placebo‐controlled, phase II study, in postmenopausal women with HR+, HER2− BC resistant to treatment with an AI in the adjuvant or metastatic setting, found that the addition of the PI3K inhibitor pictilisib to fulvestrant did not significantly improve PFS (6.6 months vs 5.1; hazard ratio, 0.74; 95% CI, 0.52‐1.06; *P* = 0.096).^51^ However, toxicity issues limited pictilisib dosing, thereby potentially limiting its efficacy.

Another randomized, phase III clinical trial, BELLE‐2 (NCT01610284), was designed to assess the efficacy of the PI3K inhibitor, buparlisib, plus fulvestrant.^50^ Postmenopausal women (aged >18 years) with histologically confirmed HR+ and HER2− inoperable locally advanced or metastatic BC with disease progression on or after AI treatment were included. A modest benefit in terms of PFS was observed, with a median PFS of 6.9 months in the combination arm vs 5.0 months in the fulvestrant alone arm (hazard ratio, 0.78; 95% CI, 0.67‐0.89; *P* < 0.0002). The safety profile of the combination was characterized by transaminitis, hyperglycemia, rash and mood disorders, especially, depression (26.2% of patients with buparlisib plus fulvestrant vs 8.9% with fulvestrant alone).^50^

BELLE‐3 (NCT01633060), which was a randomized, double‐blind, placebo‐controlled, phase III trial, included patients who were HR+/HER2−, AI‐treated, locally advanced BC that had either progressed on treatment or after treatment with everolimus. In all, 432 patients were randomized (2:1) to receive daily buparlisib plus fulvestrant or placebo plus fulvestrant.^73^ Median PFS for patients in the buparlisib arm was 3.9 months compared with 1.8 months for those in the placebo arm (*P* < 0.001). Among patients with PIK3CA mutations, PFS was 4.7 months for those in the buparlisib arm compared with 1.4 months for those in the placebo arm (*P* < 0.001).^52^

SANDPIPER (NCT02340221), which was a double‐blind, placebo‐controlled, randomized phase III study, assessed the combination of taselisib plus fulvestrant in HR+/HER2−, *PIK3CA*‐MUT locally advanced or metastatic BC postmenopausal patients with disease recurrence or progression during or after an AI. The taselisib arm demonstrated a significant improvement in investigator assessed‐PFS as confirmed by blinded independent central review‐PFS compared with the placebo arm (median 5.4 vs 7.4 months; hazard ratio, 0.70; *P* = 0.003). As per the safety profile, diarrhea (12%) was the most common grade ≥3 adverse event in the taselisib combination arm, followed by hyperglycemia (10%), colitis (3%), and stomatitis (2%). Compared with placebo, adverse events led to more taselisib discontinuations (17% vs 2%) and dose reductions (37% vs 2%).^74^ Owing to these safety concerns, further development of taselisib has been halted.^75^

Although median PFS results were promising, broader PI3K inhibition was associated with a challenging toxicity profile in patients with PIK3CA‐mutant vs wild‐type tumors.^50^, ^73^ Therefore, selective targeting of a single PI3K isoform might reduce adverse effects. To further strengthen this hypothesis, a recent phase 1a study of alpelisib (BYL719), an oral, α‐specific PI3K inhibitor demonstrated encouraging treatment response along with tolerable safety profile in patients with PIK3CA‐altered solid tumors.^76^ A phase 1b clinical trial assessing the combination of alpelisib plus fulvestrant showed manageable safety profile in patients with ER + advanced BC, and data suggest that this combination may have greater clinical activity in PIK3CA‐altered vs wild‐type tumor.^77^ Another phase 1b study using combination of letrozole and alpelisib also showed higher clinical benefit in patients with PIK3CA‐mutated tumors.^78^

A recent phase III, SOLAR‐1 trial (NCT02437318) evaluating the combination of α‐PI3K inhibitors (alpelisib) with fulvestrant in patients with PIK3CA mutations has achieved its primary endpoint (PFS). Alpelisib arm has shown significant improvement in median PFS compared to the placebo arm (11.0 months vs 5.7 months; hazard ratio, 0.65; 95% CI, 0.50‐1.25, *P* = 0.00065) at a median follow‐up of 20.0 months. It was seen that 36% patients with measurable PIK3CA‐mutated advanced BC responded to alpelisib combination whereas ORR was 16% in the placebo group (*P* = 0.0002).^79^ Further data on other endpoints from this trial is awaited and will help strengthen treatment strategy based on patient's tumor genomic profile (Table 3).

**Table 3:** Key ongoing phase III clinical trials combining alpha‐PI3K inhibitors with fulvestrant

Study name (ClinicalTrials.gov identifier)	Study arms	Study population	Outcome measures
SOLAR‐1 (NCT02437318)	Alpelisib + fulvestrant vs fulvestrant + placebo	Men and postmenopausal women with HR+/HER2 ‐ advanced BC, who received prior treatment with an AI either as (neo)‐adjuvant or for advanced disease Relapsed with evidence of progression within or more than 12 months from completion of (neo)‐adjuvant therapy Newly diagnosed advanced BC relapsed with documented progression on or after one line of endocrine therapy	PFS, OS, ORR, CBR, and QOL

AI: aromatase inhibitor; CBR: clinical benefit rate; HER2: human epidermal growth factor receptor 2; HR: hormone receptor; OS: overall survival; ORR: overall response rate; PFS: progression‐free survival; QOL: quality of life.

### 6.3 Fulvestrant in combination with mTOR inhibitors

The PI3K/AKT/mTOR pathway is a prototypic survival pathway that is constitutively activated in many types of cancer (Figure 2).^80^

The combination of everolimus, the first mTOR inhibitor introduced into clinical practice, and endocrine therapy represents an important strategy to overcome resistance.^61^ The multicenter phase II PrECOG 0102 study (NCT01797120) was designed to evaluate the combination of everolimus with fulvestrant vs fulvestrant single agent as a second‐line therapy in women with HR+/HER2− advanced BC previously treated with an AI for metastatic disease or relapsing on adjuvant AI.^81^ Kornblum et al reported a statistically significant improvement in median PFS for the addition of everolimus to fulvestrant from 5.1 to 10.3 months (hazard ratio, 0.61; 95% CI, 0.40‐0.92; stratified log‐rank *P* = 0.020). The combination was associated with greater toxicity; wherein the most frequent adverse events were oral mucositis (53%), fatigue (42%), rash (38%), anemia (31%), diarrhea (23%), hyperglycemia (19%), hypertriglyceridemia (17%), and pneumonitis (17%).^81^ The tolerability profile of both drugs was consistent with that seen in other studies.^30^, ^82^ Nonetheless, the PrECOG 0102 trial results need further confirmation by larger studies.

On the contrary, results from a very recent trial, MANTA (NCT02216786), an investigator‐led, randomized, open‐label phase II trial failed to demonstrate any benefit of adding target of rapamycin complex 1/2 (TORC1/2) inhibitor, vistusertib (AZD2014), to fulvestrant.^83^. A total of 333 patients were randomized to receive fulvestrant (n = 66), fulvestrant + vistusertib (n = 106, continuous), fulvestrant + vistusertib (n = 95, intermittent); and fulvestrant + everolimus (n = 64). Median PFS was 4.6 months (95% CI, 3.4‐6.9) in patients assigned to fulvestrant; 7.5 months (95% CI, 5.6‐9.4) in those assigned to fulvestrant + vistusertib (continuous); 7.6 months (95% CI, 5.5‐9.6) in those assigned to fulvestrant + vistusertib (intermittent); and 12.2 months (95% CI, 7.5‐14.3) in those assigned to fulvestrant + everolimus. No significant difference was recorded between the patients assigned to fulvestrant + vistusertib (continuous) and fulvestrant (hazard ratio, 0.87; 95% CI, 0.62‐1.23; log‐rank *P* = 0.420); fulvestrant + vistusertib (intermittent) and fulvestrant (hazard ratio, 0.78; 95% CI, 0.55‐1.12; log‐rank *P* = 0.16); and fulvestrant + vistusertib (continuous) and fulvestrant + vistusertib (intermittent) (hazard ratio, 1.11; 95% CI, 0.81‐1.52; log‐rank *P* = 0.520). PFS was significantly longer in patients assigned to fulvestrant + everolimus compared with fulvestrant + vistusertib (continuous) (hazard ratio, 0.64; 95% CI, 0.45‐0.91; log‐rank *P* = 0.010) and fulvestrant + everolimus compared with fulvestrant (hazard ratio, 0.64; 95% CI, 0.43‐0.94; log‐rank *P* = 0.020).^83^ As reported in Table 4, results from an ongoing trial are awaited.

**Table 4:** Ongoing phase II clinical trial combining mTOR inhibitors with fulvestrant

Study name (ClinicalTrials.gov identifier)	Study arms	Study population	Outcome measures
NCT02049957	MLN0128(Dual mTORC1/2 Inhibitor) + Fulvestrant	Postmenopausal women with HR+/HER2− advanced or metastatic BC that has progressed on treatment with everolimus in combination with exemestane or fulvestrant	Percentage of patients experiencing AEs, CBR, ORR, and PFS

AE, adverse event; BC, breast cancer; CBR, clinical benefit rate; HER2, human epidermal growth factor receptor 2; HR, hormone receptor; ORR, overall response rate; PFS, progression‐free survival.

### 6.4 Fulvestrant in combination with endothelial growth factor receptor and HER2 inhibitors

Given the overexpression ofendothelial growth factor receptor or HER2 in some HR + BC, it has been suggested that EGFR‐ and HER2‐targeting agents could be a potential treatment strategy.^84^, ^85^ The phase III trial Cancer and Leukaemia Group B 40302B (CALGB 40302/Alliance) study (NCT00390455) investigated the combination of the dual EGFR‐HER2 inhibitor lapatinib with fulvestrant compared with lapatinib alone in women with advanced HR+/HER2− or HER2 + BC that was resistant to endocrine therapy. The study reported no benefit from the addition of lapatinib to fulvestrant in either PFS (4.7 vs 3.8 months, hazard ratio, 1.04, *P = *0.370), or OS (30 vs 26.4 months, hazard ratio, 0.91, *P* = 0.250). Similarly, no significant improvement in PFS (5.9 vs 3.3 months; *P* = 5.530) was observed in the HER2+ subgroup of patients.^54^

### 6.5 Fulvestrant in combination with vascular endothelial growth factor receptor and epidermal growth factor receptor tyrosine kinase inhibitors (TKIs)

A phase II placebo‐controlled trial assessed the addition of vandetanib to fulvestrant (n = 61) compared with placebo (n = 68) in postmenopausal women with bone‐only or bone‐predominant HR + metastatic BC (NCT00811369). Turnover of bone biomarker, urine N‐telopeptide (uNTx), was used to assess whether vandetanib improved uNTx response when added to fulvestrant in patients with bone metastases. No significant difference was detected between groups for PFS (hazard ratio, 0.95, 95% CI, 0.65‐1.38) or OS (hazard ratio, 0.69, 95% CI, 0.37‐1.31). In addition, investigators also concluded that adding vandetanib to fulvestrant did not result in an improvement of biomarker response, PFS, or OS in patients with bone metastases.^87^

## 7 RATIONALE FOR TREATMENT STRATEGY

With the evolving therapeutic advancements in treating BC, clinicians now have multiple strategies to opt from. Care should be taken to determine whether all patients who are suitable for endocrine therapy should receive monotherapy or combination therapy. Even if first‐line trials demonstrate benefit in terms of OS, questions as to whether similar effects can be achieved with crossover remains unanswered. CDK4/6 inhibitors show superior efficacy compared with endocrine monotherapy; however, the advantage over OS is yet to be reported.^44^, ^64^ Therefore, it is reasonable to still consider single‐agent fulvestrant therapy for some patients, ideally for those at low risk with low activity and presumed sensitivity. Unfortunately, there is a great deal of heterogeneity in HR + metastatic BC,^88^ and thus, there is a critical need for developing predictive biomarkers to allow improved guidance in treatment choice.^89^ When recommending appropriate endocrine therapy, fulvestrant's efficacy must be weighed against its intramuscular administration,^90^ which necessitates more frequent visits. On the contrary, a potential advantage of fulvestrant in terms of improved treatment compliance due to its monthly parenteral administration compared with daily oral intakes of other endocrine therapies should be acknowledged. A particular benefit with fulvestrant has been seen in those with non‐visceral disease and *ESR1* mutation status.^91^ Clinically, impressive response rates have also been reported with the combination of CDK4/6 with fulvestrant, with objective response rates in excess of 40%^63^, ^92^; which is in the range of chemotherapy response rates in phase III trials with endocrine receptor‐positive disease.^93^, ^94^ Owing to these qualities, combination strategies especially with CDK4/6 inhibitors seem to offer more benefits from endocrine therapy than chemotherapy.

The choice of endocrine monotherapy can be influenced by *ESR1* mutational status and disease pattern (non‐visceral vs visceral disease). As the status of *ESR1* mutations impacts the outcome of patients in response to endocrine therapy,^91^ detecting *ESR1* mutations may be a promising method of individualizing treatment for metastatic BC.^95^ Retrospective analyses from completed clinical trials suggest that these mutations are prognostic and predictive of resistance to an AI in metastatic disease.^96^, ^97^ However, prospective studies to confirm these results and to determine the best treatment combinations for patients with *ESR1* mutations are required. Higher doses of fulvestrant could improve outcomes for patients with these mutations, which are mostly preclinical.^98^ Lack of robust and reliable biomarkers to choose a specific combination therapy strategy is of serious concern. Although targeting PI3K pathway seems a feasible option, efficacy and safety concerns around PI3K inhibitors do not allow their use in clinical setting.

Guidance on selecting and sequencing of treatments should be reevaluated following the availability of data for both OS and PFS, from the FALCON, PALOMA 2, MONALEESA 2, MONALEESA 3, and MONARCH 3 trials.^30^, ^47^, ^99^, ^100^ Based on previously available data, key recommendations for endocrine therapy in HR + metastatic BC from the National Comprehensive Cancer Network (NCCN) and American Society for Clinical Oncology have been made. Both guidelines recommend offering hormonal therapy in patients with tumors and any level of HR expression and that therapy decision must consider the type of adjuvant treatment, disease‐free interval, and extent of disease at the time of recurrence. It also recommends that treatment should be continued until disease progression occurs and that endocrine therapy and chemotherapy should not be combined.^5^, ^102^ Perhaps results from few ongoing trials such as PADA‐1 and SONIA may shed some light on optimal combination therapies in improving OS in BC patients.

## 8 FULVESTRANT AS FIRST‐LINE THERAPY

Benefits of endocrine therapy in treating HR + BC are well recorded. Despite these interventions in the adjuvant setting, ~40% to 50% of HR+ patients relapse.^103^ As per the third International Consensus Conference for Advanced BC (ABC 3) guidelines, endocrine resistance includes patients whose disease relapsed while on the first 2 years of adjuvant endocrine therapy, or disease progression within first 6 months of first‐line endocrine therapy for BC, while still on endocrine therapy. Accordingly, studies in such resistant patients have shown promising effects of combination therapy with fulvestrant,^47^ either as a first‐line treatment option or as a second‐line treatment option. However, choice of first‐line or second‐line endocrine therapy should take into consideration few other key points such as symptoms, extent of disease, prior agent exposure, and response to previous hormone therapy. Considering these points, a clinician should take a call on whether or not to opt for monotherapy or combination therapy based on type of patient (resistant or sensitive).The efficacy of fulvestrant in the first‐line setting, either as monotherapy or in combination with anastrozole, in endocrine‐naïve patients has been supported by findings from clinical studies.^27^, ^28^, ^31^ Briefly, fulvestrant monotherapy or in combination with anastrozole have been demonstrated to be effective and safe for the initial treatment of postmenopausal women with advanced HR+/HER2− BC.^41^ Fulvestrant 500 mg is now approved first‐line monotherapy, based on the findings from the FALCON study, in USA, Europe, Japan, and Russia.

Given the numerous BC subtypes and therapies, selecting first‐line therapy for postmenopausal HR+/HER2− advanced BC remains complex and challenging. Additionally, increased use of AI therapy in the adjuvant setting has further complicated this situation.^104^ However, at the initiation of first‐line endocrine therapy, the hardest question is whether or not to use monotherapy or combination therapy. Both single‐agent therapy (AI, tamoxifen, and fulvestrant) and the combination of different agents (endocrine therapy plus other endocrine agent, or endocrine therapy in combination with a targeted agent) are reasonable alternatives.

Additionally, other first‐line treatment options include the combination of a CDK4/6 inhibitor, such as palbociclib, abemaciclib, or ribociclib, with an AI. Data from one phase II and three phase III clinical trials have demonstrated that adding a CDK4/6 inhibitor (palbociclib or ribociclib) to letrozole results in significant improvements in PFS vs an AI.^41^, ^99^, ^100^ Furthermore, recent evidence from the MONALEESA‐3 trial has also supported and emphasized on combining CDK4/6 inhibitor (ribociclib) with fulvestrant as a first‐line treatment option for HR+/HER2− advanced BC. In summary, results from the PALOMA‐3, MONARCH‐2, and MONALEESA‐3 trials have consistently proven that the combination of CDK4/6 inhibitors with fulvestrant to be efficient in improving PFS in resistant patients with relapse patients after first‐line endocrine therapy in advanced BC.

Fulvestrant monotherapy could also be a treatment option in low‐risk patients, with very limited, bone‐only, or with non‐visceral disease. Furthermore, fulvestrant monotherapy might be a choice for patients with comorbidities and for those unable to tolerate combination targeted therapy with an eventually higher rate of myelosuppression or in situations where targeted therapies are not available.^4^

## 9 FULVESTRANT AS SECOND‐LINE THERAPY

Several treatment options exist for second‐line therapy: thus, single‐agent therapy (fulvestrant) and the combination of fulvestrant plus a targeted agent (mTOR or CDK 4/6 inhibitor) could be considered. For second‐line monotherapies, nonsteroidal AI exemestane and fulvestrant proved to be equally effective.^105^ The use of fulvestrant 500 mg as monotherapy in second‐line treatment has been supported by the evidence provided by the CONFIRM study.^24^, ^25^, ^106^ Fulvestrant received a new indication in 2016, 2017, and 2018^35^, ^69^, ^107^ with the approval of the combination with CDK 4/6 inhibitors, palbociclib, abemaciclib, and ribociclib based on PALOMA 3, MONARCH 2, and MONALEESA 3 trials, respectively.^44^, ^46^, ^47^ In pre/perimenopausal patients, palbociclib/abemaciclib plus fulvestrant in combination with ovarian‐function suppression, is recommended.^44^, ^45^Recently, results for the combination of fulvestrant plus everolimus became available^108^, ^109^; however, the combination has not been approved and has not been introduced into clinical practice.

In summary, data on efficacy and tolerability support the use of the second‐line therapy fulvestrant as a monotherapy or in combination with the CDK4/6 inhibitors, abemaciclib, ribociclib, and palbociclib.^46^, ^47^, ^64^, ^69^ In the context of the available treatment choices (eg, monotherapy or combination), decisions may be channeled considering the adverse event profiles of the drugs, patient performance status, comorbidities, and patient preferences.

## 10 CONCLUSION

Fulvestrant, with its unique mode of action, has showed efficacy in treating patients with HR+/HER2− advanced BC, alone or in combination with other endocrine agents or targeted therapies. The combination of fulvestrant with other targeted therapies is emerging as a therapeutic choice for patients who need a well‐tolerated therapy, and it also offers a balance of efficacy, safety, and quality of life.

Fulvestrant monotherapy shows superior efficacy as first‐line treatment option, especially in endocrine‐naïve cases, while combining fulvestrant with a CDK4/6 inhibitor could be the preferred treatment option in patients with prior exposure to an AI. Identifying biomarkers will lead to a more accurate selection of patients likely to benefit from fulvestrant monotherapy or from existing combinations. Although well‐defined indications for fulvestrant in the therapeutic algorithm of advanced HR + BC does exist, the optimal position has not been clearly defined. Several research strategies to evaluate the potential of fulvestrant in advanced BC are ongoing.

## CONFLICT OF INTEREST

None declared.
